# Model-Based Assessment of C-Peptide Secretion and Kinetics in Post Gastric Bypass Individuals Experiencing Postprandial Hyperinsulinemic Hypoglycemia

**DOI:** 10.3389/fendo.2021.611253

**Published:** 2021-03-15

**Authors:** Michele Schiavon, David Herzig, Matthias Hepprich, Marc Y. Donath, Lia Bally, Chiara Dalla Man

**Affiliations:** ^1^ Department of Information Engineering, University of Padova, Padova, Italy; ^2^ Department of Diabetes, Endocrinology, Nutritional Medicine and Metabolism, Inselspital, Bern University Hospital, University of Bern, Bern, Switzerland; ^3^ Division of Endocrinology, Diabetes and Metabolism, University Hospital Basel, Basel, Switzerland

**Keywords:** model identification, parameter estimation, obesity, insulin secretion, oral minimal model, OGTT, mixed meal

## Abstract

Assessment of insulin secretion is key to diagnose postprandial hyperinsulinemic hypoglycemia (PHH), an increasingly recognized complication following bariatric surgery. To this end, the Oral C-peptide Minimal Model (OCMM) can be used. This usually requires fixing C-peptide (CP) kinetics to the ones derived from the Van Cauter population model (VCPM), which has never been validated in PHH individuals. The objective of this work was to test the validity of the OCMM coupled with the VCPM in PHH subjects and propose a method to overcome the observed limitations. Two cohorts of adults with PHH after gastric bypass (GB) underwent either a 75 g oral glucose (9F/3M; age=42±9 y; BMI=28.3±6.9 kg/m^2^) or a 60 g mixed-meal (7F/3M; age = 43 ± 11 y; BMI=27.5±4.2 kg/m^2^) tolerance test. The OCMM was identified on CP concentration data with CP kinetics fixed to VCPM (VC approach). In both groups, the VC approach underestimated CP-peak and overestimated CP-tail suggesting CP kinetics predicted by VCPM to be inaccurate in this population. Thus, the OCMM was identified using CP kinetics estimated from the data (DB approach) using a Bayesian Maximum a Posteriori estimator. CP data were well predicted in all the subjects using the DB approach, highlighting a significantly faster CP kinetics in patients with PHH compared to the one predicted by VCPM. Finally, a simulation study was used to validate the proposed approach. The present findings question the applicability of the VCPM in patients with PHH after GB and call for CP bolus experiments to develop a reliable CP kinetic model in this population.

## Introduction

Postprandial hyperinsulinemic hypoglycemia is an increasingly recognized metabolic complication affecting up to a third of patients following gastric bypass surgery (1, 2). While the underlying mechanisms remain to be fully elucidated (3), excessive postprandial insulin exposure due to exaggerated insulin secretion and/or diminished insulin clearance are key pathophysiological hallmarks of postprandial hyperinsulinemic hypoglycemia (4–6). Thus, reliable estimation of insulin secretion is fundamental to improve our understanding and diagnostic armamentarium of this complex condition.

Insulin secretion is not directly measurable *in vivo* but can be reconstructed from plasma C-peptide concentrations using non-parametric, e.g., deconvolution (7), or parametric approaches, e.g., structural models (8–11). Nevertheless, both approaches require the knowledge of C-peptide kinetics, usually described by a linear two-compartment model (12).

The direct measurement of C-peptide kinetics requires the injection of a C-peptide bolus under somatostatin infusion to block its endogenous secretion (13). However, to reduce patient burden and costs associated with additional experiments, C-peptide kinetic parameters predicted by the Van Cauter population model (14) can be used. The Van Cauter population model was originally validated in normal, obese, and non-insulin-dependent diabetic individuals and its use within approaches to estimate insulin secretion was shown to yield similar average estimates in the target population as when individual estimates from C-peptide bolus data are used (15, 16). However, this might not hold true when the Van Cauter population model is applied to different populations, such as patients having undergone bariatric surgery, procedures that substantially alter glucose kinetics and secretion of gluco-regulatory hormones (17). Since inaccuracy in C-peptide kinetics may negatively affect the estimation of insulin secretion, the applicability of the Van Cauter population model to predict C-peptide kinetics in patients suffering from postprandial hyperinsulinemic hypoglycemia must be investigated.

The aim of this work was to assess the validity of the Van Cauter population model in post-gastric bypass individuals suffering from postprandial hyperinsulinemic hypoglycemia. This was done by coupling it with a model for the estimation of C-peptide secretion. Among the models proposed in the literature, here the so-called Oral C-peptide Minimal Model (OCMM) (11, 18) was used. In a second step, a new methodology was proposed to overcome the observed limitations when using the Van Cauter population model in these subjects. Finally, the validity of this new methodology was tested by means of an *in silico* experiment.

## Database and Methods

### Databases

Data from twenty-two post-gastric bypass individuals suffering from postprandial hyperinsulinemic hypoglycemia gathered during two separate clinical trials were used in this work.

Twelve subjects (Cohort 1 - OGTT) (9F; age = 42±9 y; BMI = 28.3±6.9 kg/m^2^) were studied at the University Hospital Bern, Bern, Switzerland (NCT03609632). Participants arrived at the clinical research facility at 0800 after an overnight fast. An intravenous cannula was inserted in one arm for blood sampling and kept open with a saline infusion. Participants underwent a standard oral glucose tolerance test (OGTT) consisting in the ingestion of 75 g of dextrose and the frequent sampling for plasma glucose, insulin and C-peptide concentrations for 210 min after glucose ingestion. Samples were taken every 15 min until 60 min after glucose ingestion and every 30 min subsequently. Hypoglycemia, defined as plasma glucose level < 2.8 mmol/L, was treated using intravenous dextrose (10%) to reach euglycaemia. Plasma glucose was determined from venous blood using the Biosen C-line analyser (IGZ Instruments AG, Zurich, Switzerland). Insulin and C-peptide concentration were measured by conventional immunoassays (Roche Diagnostics, Mannheim, Germany).

The other 10 subjects (Cohort 2 - MMTT) (7F; age = 43±11 y; BMI = 27.5±4.2 kg/m^2^) were studied at the University Hospital Basel, Basel, Switzerland (NCT03200782). Subjects participated in a double-blind, double-dummy placebo controlled, randomized, cross-over trial where each subject underwent a standardized liquid mixed-meal tolerance test (MMTT, 300 ml Ensure plus®, Abbott, 60 g carbohydrates, 450 kcal) on three occasions receiving either a placebo, a SGLT2-inhibitor or a IL-1 receptor agonist. For the purpose of this work, only data from the placebo visit were used. More details on the study protocol can be found in (19). Plasma glucose, insulin and C-peptide concentrations were sampled every 30 min for 180 min after mixed-meal ingestion. In case of symptomatic hypoglycemia, defined by the Whipple’s triad with plasma glucose < 2.5 mmol/L, immediate glucose measurement and blood sampling was performed followed by the administration of 10 g glucose (orally or intravenously).

**Figure 1:**
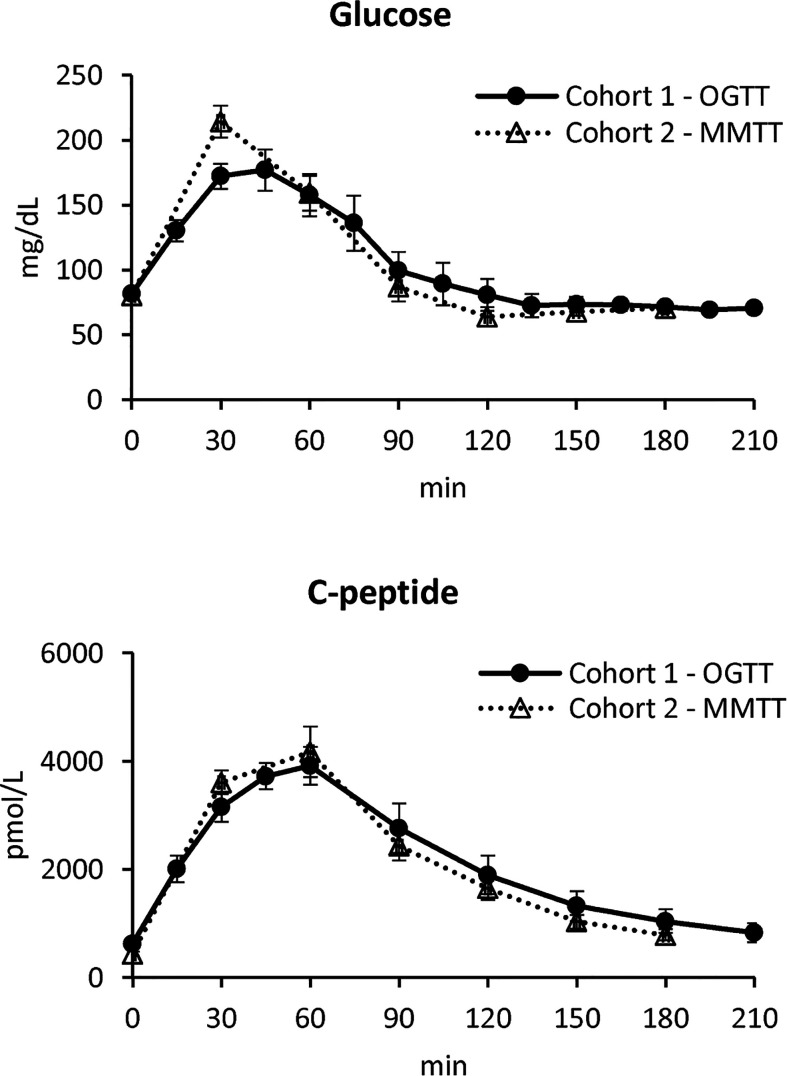
Plasma glucose (top) and C-peptide (bottom) concentrations in Cohort 1 – OGTT (continuous line with black circles) and Cohort 2 – MMTT (dotted line with triangles) (mean ± SE).

In **Figure 1**, mean ± standard error (SE) of plasma glucose (top) and C-peptide (bottom) concentrations of the two studies are reported.

### Methods

#### The Oral C-Peptide Minimal Model (OCMM)

**Figure 2:**
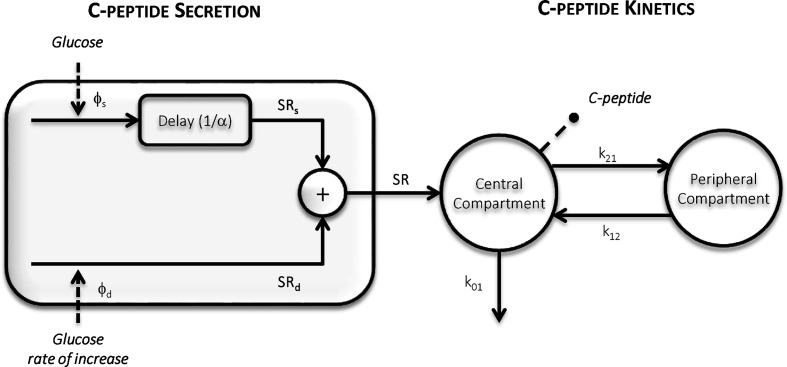
The Oral C-peptide Minimal Model (OCMM) (11, 18) with its secretion (left) and kinetics (right) components. SR is the pancreatic secretion, Φ_s_ and Φ_d_ static and dynamic β-cell responsivity indices respectively, 1/α time constant of the static component, and k_01_, k_12_, and k_21_ C-peptide kinetic parameters.

The oral C-peptide minimal model (OCMM, **Figure 2**) (11, 18) interprets plasma C-peptide concentration in relation to the observed changes in glucose concentration and provides a quantification of β-cell responsivity to glucose.

C-peptide kinetics are described by the well-known two-compartment model (12) (**Figure 2**, right panel):

(1) $$\{\begin{array}{ll} C{\dot{P}}_{1}(t)=-(k_{01}+k_{21})\cdot CP_{1}(t)+k_{12}\cdot CP_{2}(t)+SR(t) & CP_{1}(0)=0\\ C{\dot{P}}_{2}(t)=-k_{12}\cdot CP_{2}(t)+k_{21}\cdot CP_{1}(t) & CP_{2}(0)=0 \end{array}$$

where CP_1_ and CP_2_ (pmol/L) are the above-basal C-peptide concentrations in the first (central) and second (peripheral) compartments respectively, k_ij_ (min^-1^) the C-peptide kinetic parameters, with k_01_ representing the C-peptide fractional metabolic clearance rate (MCR, min^-1^) and SR (pmol/L/min) the above basal pancreatic secretion normalized by the volume of distribution of the first compartment.

SR is modeled as the sum of two components controlled by glucose concentration (static component, SR_s_) and its rate of increase (dynamic component, SR_d_) (**Figure 2**, left panel):

(2) $$SR(t)=SR_{s}(t)+SR_{d}(t)$$

In particular, SR_s_ represents the provision of new releasable insulin Y(t) (pmol/L/min):

(3) $$SR_{s}(t)=Y(t)$$

which is controlled by glucose concentration (G, mmol/L) according to:

(4) $$\dot{Y}(t)=-\alpha \left[Y(t)-\beta \cdot [G(t)-h]\right]Y(0)=0$$

Thus, SR_s_ is dynamically related to glucose concentration and tends toward a steady-state value with a time constant 1/α (min). The steady-state value is linearly dependent from glucose concentration above a threshold level h (mmol/L), here fixed to pre-meal (basal) glucose level G_b_ (20), through a parameter β (min^-1^). SR_d_ represents the secretion of promptly releasable insulin and is proportional, through a parameter k_d_ (dimensionless), to the rate of glucose increase:

(5) $$SR_{d}(t)=\{\begin{array}{ll} k_{d}\cdot \dot{G}(t) & if\;\dot{G}(t)>0\\ 0 & if\;\dot{G}(t)\leq 0 \end{array}$$

Basal insulin secretion (SR_b_) can be calculated as:

(6) $$SR_{b}=k_{01}\cdot CP_{1b}$$

where CP_1b_ is the basal C-peptide concentration in the first compartment.

From model parameters, indices of static (Φ_s_ = β, 10^-9^ min^-1^), dynamic (Φ_d_ = k_d_, 10^-9^) and basal β-cell responsiveness (Φ_b_ = SR_b_/G_b_, 10^-9^ min^-1^) can be derived. Finally, an index of total β-cell responsiveness to glucose (Φ_tot_, 10^-9^ min^-1^) (11) can be calculated as:

(7) $$\phi_{tot}=\frac{\int_{0}^{T}[SR(t)+SR_{b}]dt}{\int_{0}^{T}G(t)dt}=\frac{\phi_{d}\cdot (G_{max}-G_{b})+\phi_{s}\cdot \int_{0}^{T}[G(t)-h]dt+T\cdot \phi_{b}\cdot G_{b}}{\int_{0}^{T}G(t)dt}$$

where T (min) is the time at which the system is assumed to return to steady-state conditions after the perturbation (here assumed T=300 min).

#### Model Identification

The OCMM is *a priori* uniquely identifiable if the measured C-peptide data are assumed as model output and the measured glucose concentrations as known input (21, 22). Parameters were estimated with a Bayesian Maximum a Posteriori (MAP) estimator (23), which requires the maximization of the *a posteriori* probability density function of the parameter vector ***p*** *= [k_d_, α, β, k_01_, k_12_, k_21_]*:

(8) $${\hat{p}}_{MAP}=arg\underset{p}{\max}f_{p|z}(p|z)$$

which, by recalling the Bayes theorem, can be rewritten as

(9) $${\hat{p}}_{\mathrm{MAP}}=arg\underset{p}{\max}\frac{f_{z|p}(z|p)f_{p}(p)}{f_{z}(z)}$$

where *f_**z**_*_|_ *_**p**_* (***z***|***p***) is the likelihood of the data, *f_z_*(***z***) is the probability density function of the data vector ***z*,** which can be ignored in the maximization problem since it does not depend on ***p***, and *f_**p**_*(***p***) is the *a priori* probability density function of ***p.*** The definition of *f_**p**_*(***p***) differs depending on the adopted identification approach, as detailed below.

##### C-Peptide Kinetics Fixed to Van Cauter Population Model (VC Approach)

Here, we assumed ***p*** *=[* ***p_1,_ p_2_*** *]* with ***p_1_*** *=[k_d_, α, β]* and ***p_2_*** *=[k_01_, k_12_, k_21_].* The *a priori* probability density function *f_p_*_1_ **(***p***_1_) was assumed to be noninformative for all the parameters except for α to improve the numerical identifiability of the model (11), especially when a limited number of samples is available. The C-peptide kinetic parameters (***p_2_*)** were fixed to those predicted by the Van Cauter (VC) population model (**Table 1**) (14). In that study, a method was proposed to estimate the kinetics parameters of the two-compartment model of C-peptide kinetics (12) from patient demographics. The model was originally validated in a large database of normal, obese, and non-insulin-dependent individuals with diabetes. However, it has never been validated in PHH subjects.

**Table 1:** Procedure to obtain the c-peptide kinetic parameters using the Van Cauter population model (14).

**Step 1:**	Determine subject’s type: normal, obese, non-insulin dependent diabetes (NIDDM)				
**Step 2:**	Determine C-peptide short half-life (*a*) and fraction (*f*) parameters				
			*Normal*	*Obese*	*NIDDM*
	Short half-life (*a*)	[min]	4.95	4.55	4.52
	Fraction (*F*)	[dimensionless]	0.76	0.78	0.78
**Step 3:**	Derive the long half-life (*b*) parameter according to the equation				
	Long half-life (*b*)	[min]	0.14·(*age* [*year*] + 29.2)		
**Step 4:**	Determine the C-peptide kinetic parameters as follows				
	*k_12_*	[min^-1^]	*F*·(ln(2)/*b*) + (1–*F*)·(ln(2)/*a*)		
	*k_01_*	[min^-1^]	(ln(2)/*a*)·(ln(2)/*b*)/*k*_12_)		
	*k_21_*	[min^-1^]	(ln(2)/*a*) + (ln(2)/*b*) – *k*_01_ – *k*_12_		

**Figure 3:**
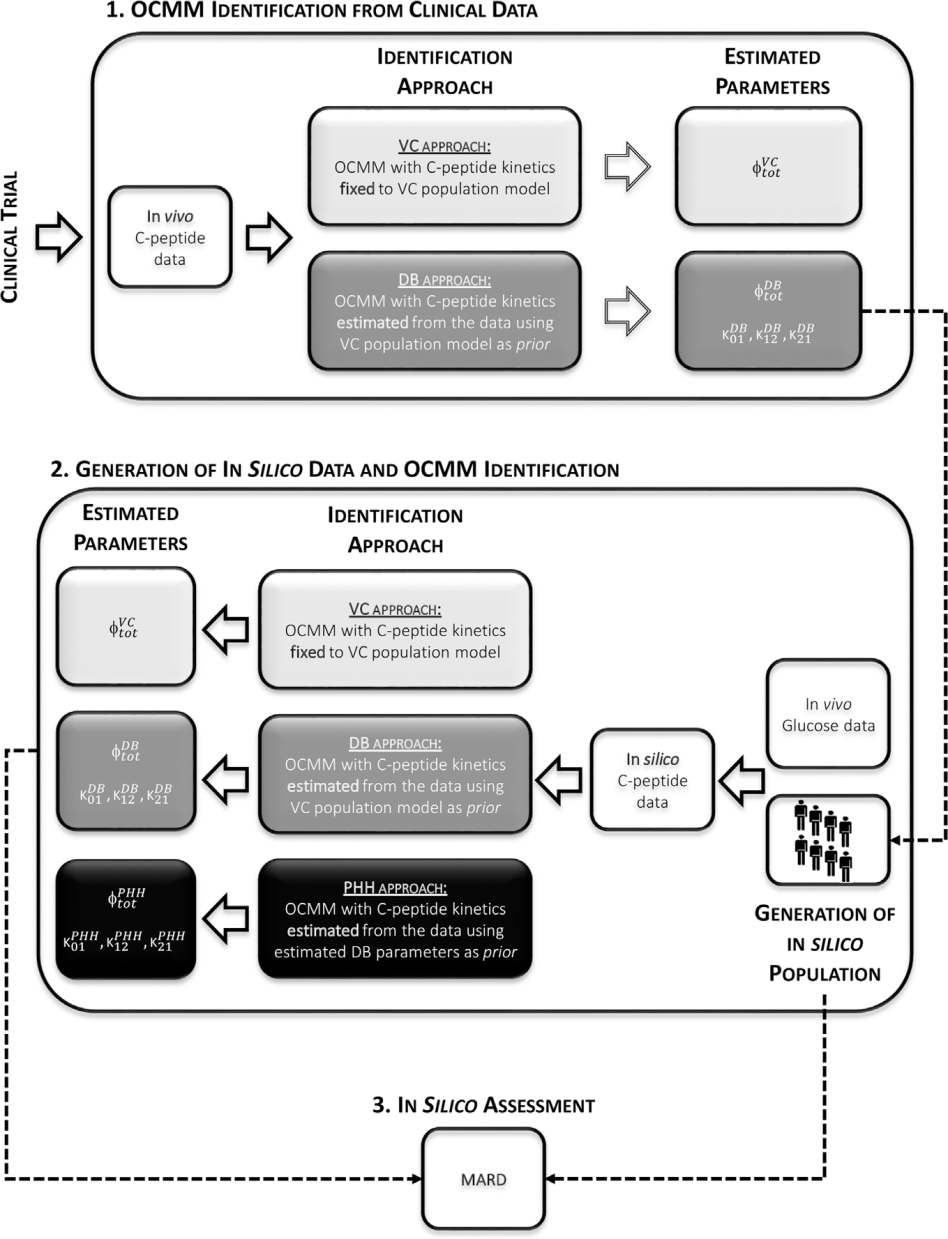
Overview of the study workflow. 1: Identification of the OCMM by both the VC and the proposed DB approach from *in vivo* data. 2: Use of estimated parameters with the DB approach to randomly generate the in *silico* population from which C-peptide concentration curves were simulated. Identification of the OCMM using the VC, DB, and PHH approach using in *silico* data. 3: Calculation of mean absolute relative difference (MARD) for the assessment of the proposed methodology using *in silico* results.

The VC approach provided unsatisfactory results in terms of model ability to predict the data in this population (see Results section). Therefore, another identification approach was tested (DB approach). Finally, a simulation study was also performed to test the accuracy of this method. An overview of the workflow is shown in **Figure 3**.

##### C-Peptide Kinetics Estimated from the Data (DB Approach)

Here, the C-peptide kinetic parameters (***p_2_***) were estimated, together with the secretion parameters (***p_1_***) from the C-peptide data (data-based, DB) using the MAP estimator of eqs. 8 and 9. The *a priori* probability density function *f_**p**_*_1_ (***p***_1_) was the same as the one adopted in the VC approach, while *f_**p**_*_2_ (***p***_2_) was derived from the Van Cauter population model (14).

For both approaches (VC and DB), measurement error of C-peptide concentration was assumed to be independent, Gaussian, with zero mean and variance dependent on C-peptide concentrations (24). Glucose concentration and its time derivative were used as error-free model inputs. Here, the time derivative of glucose concentration was calculated using a stochastic regularized deconvolution method (25), particularly suitable in case of noisy signals. The precision of model parameter estimates was quantified by its coefficient of variation (CV, %) (23). Parameter estimation and statistical analyses were carried out using Matlab^®^ (R2016a); differential equations were integrated using a method based on an explicit Runge-Kutta (4^th^–5^th^ order) pair formula implemented in the Matlab function *ode45* (26).

#### *In Silico* Assessment

The ability of the proposed DB approach to accurately estimate the kinetic and secretion parameters of the OCMM was assessed by computer simulation.

To set up the simulation, we first used the C-peptide kinetic parameters estimated with the DB approach to derive the joint C-peptide kinetic parameter distribution in our PHH subjects. Specifically, similarly to what done in (27), the kinetic parameters were assumed to be log-normally distributed with mean (***μ_p2_***) and covariance matrix (***Ʃ_p2_***):

(10) $$\mu_{p2}=\left[mean\left(\ln k_{01}\right),mean\left(\ln k_{12}\right),mean\left(\ln k_{21}\right)\right]$$

(11) $$\sum_{p2}=\left[\begin{array}{ccc} var\left(\ln k_{01}\right) & covar\left(\ln k_{01},\ln k_{12}\right) & covar\left(\ln k_{01},\ln k_{21}\right)\\ covar\left(\ln k_{01},\ln k_{12}\right) & var\left(\ln k_{12}\right) & covar\left(\ln k_{12},\ln k_{21}\right)\\ covar\left(\ln k_{01},\ln k_{21}\right) & covar\left(\ln k_{12},\ln k_{21}\right) & var\left(\ln k_{21}\right) \end{array}\right]$$

From the above distribution, we randomly extracted 1,100 kinetic parameter vectors ***p_2_*** *=[k_01_, k_12_, k_21_]*. In particular, for each of the 22 subjects in our database, 50 triplets (***p_2_***) were randomly generated and coupled to the set of estimated secretion parameters (***p_1_***) together with the corresponding glucose curve. This allowed us to create 1,100 *in silico* (virtual) subjects, with known kinetic and secretion parameters, for which the C-peptide concentration after an oral test was simulated using the subject-specific glucose curve as input signal. Such *in silico* C-peptide profiles were then sampled and corrupted by an additive Gaussian random noise with zero mean and variance as in (24). Finally, the OCMM was identified using both the VC and DB approach, as described in Section 2.2.2. In addition, we tested to what extent the final parameter estimates were affected by the choice of the *a priori* information. To do so, the model was also identified using as *prior* information the C-peptide kinetic parameters estimated with the DB approach (PHH approach).

#### Statistical Analysis

Normality of variable distributions was assessed by Lilliefors test and, since some of the variable of interest were not normally distributed, nonparametric tests were used. In particular, for the parameters estimated from *in vivo* (clinical) data, differences between the VC and the DB approaches, within the same cohort, were assessed using a Wilcoxon signed-rank test; while a Mann-Whitney-U test was used to compare cohorts (Cohort 1-OGTT vs. Cohort 2-MMTT). For the results of the *in silico* experiment, mean absolute relative differences (MARD) between the estimated parameters from the respective approach and the known parameters were calculated in order to assess the validity of the approaches. Differences in MARD among identification approaches (VC vs. DB vs. PHH) were then assessed by Kruskal-Wallis test and post-hoc analysis was performed using Dunn-Sidak correction for multiple comparison (26). Results are reported as median [25^th^, 75^th^] percentile unless otherwise specified. A p-value <0.05 was considered statistically significant.

## Results

### Model Identification

**Figure 4:**
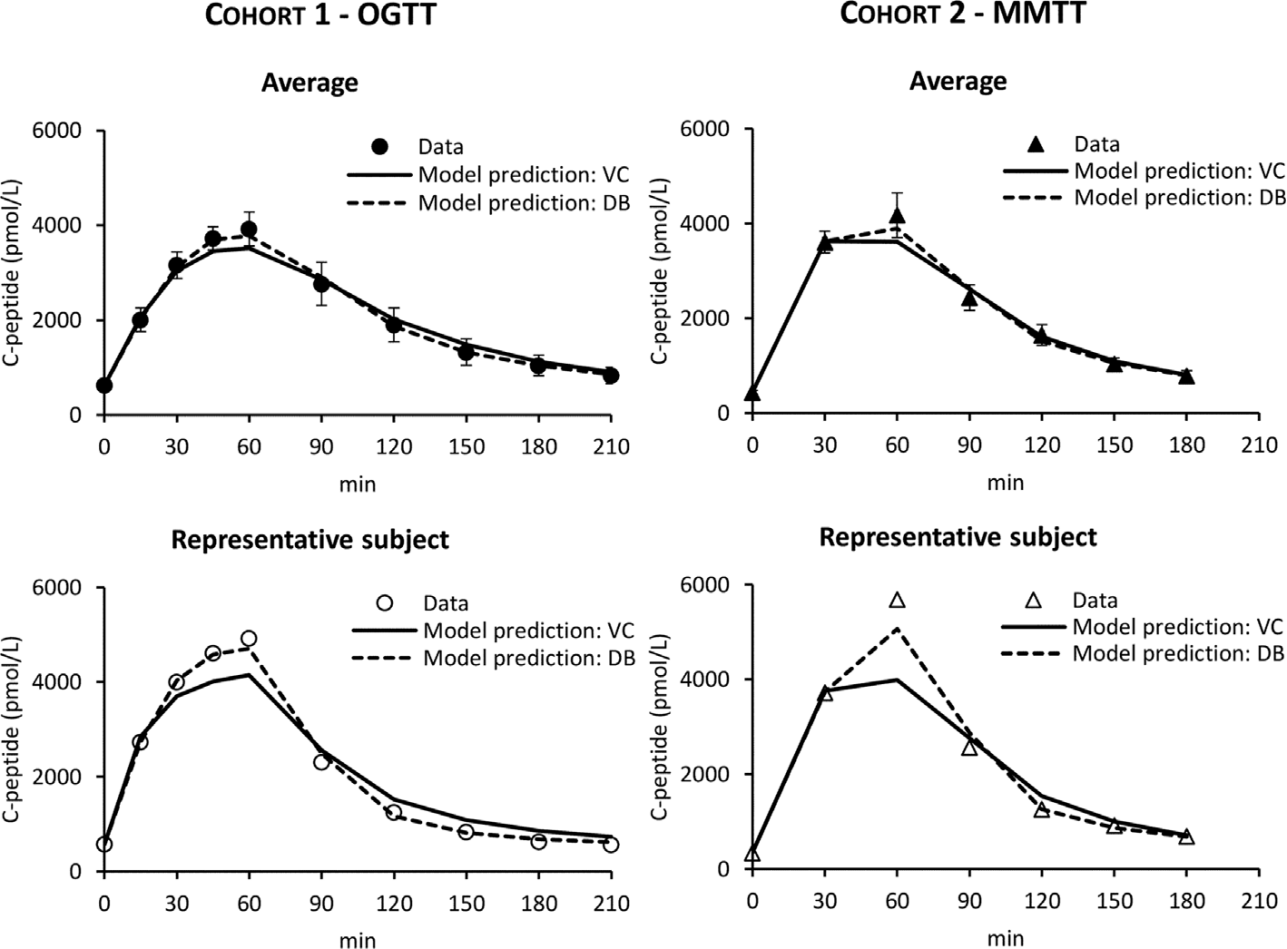
C-peptide data in Cohort 1 – OGTT (left panels, circles) and Cohort 2 – MMTT (right panels, triangles) vs. model predictions obtained with VC (continuous line) and DB (dashed line) approach. Mean ± SE are reported in the top panels while representative subjects in the bottom panels.

**Figure 5:**
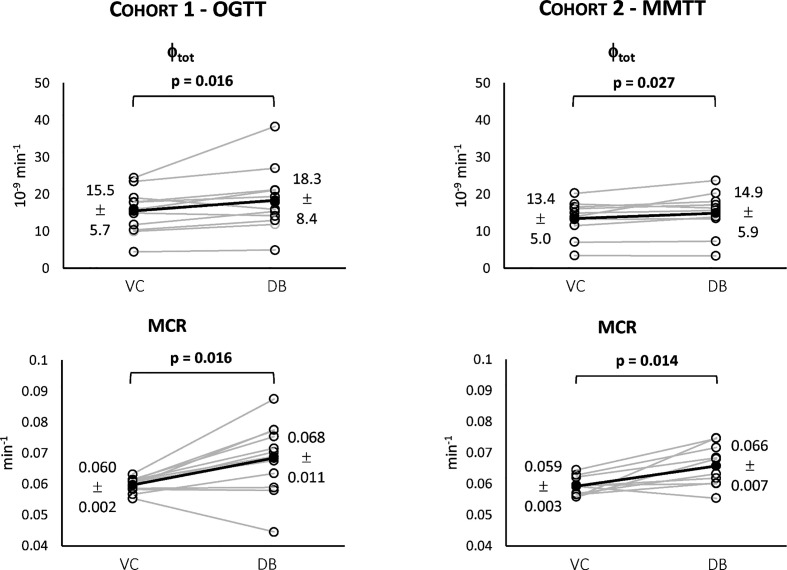
Individual estimates of the index of total β-cell responsivity to glucose (Φ_tot_, top) and C-peptide metabolic clearance rate (MCR, bottom) for both Cohort 1 – OGTT (left) and Cohort 2 – MMTT (right).

In both cohorts, the VC approach underestimated C-peptide peak and overestimated C-peptide tail, while the DB accurately predicted C-peptide data in all subjects (**Figure 4**). Individual estimates of the key parameters Φ_tot_ and MCR are reported in **Figure 5**.

For both cohorts, a statistically significant difference between the VC and the DB approach was observed for Φ_tot_ (**Figure 5**, top) and MCR (**Figure 5**, bottom). No statistically significant differences between cohorts were observed for both variables within the VC and DB approach, respectively. When pooling data from both cohorts, a significantly higher Φ_tot_ (16.1 [13.5, 20.0] 10^-9^ min^-1^ vs. 15.4 [11.6, 17.7] 10^-9^ min^-1^, p<0.01) was observed with the DB than the VC approach, which was accompanied by a significantly higher C-peptide MCR (0.068 [0.061, 0.074] min^-1^ vs. 0.059 [0.057, 0.061] min^-1^, p<0.001) in the DB vs. VC approach. All parameters were estimated with precision: CV among parameters was 19 [14, 24] % using the DB approach and 9 [5, 17] % using the VC approach.

### *In Silico* Assessment

**Figure 6:**
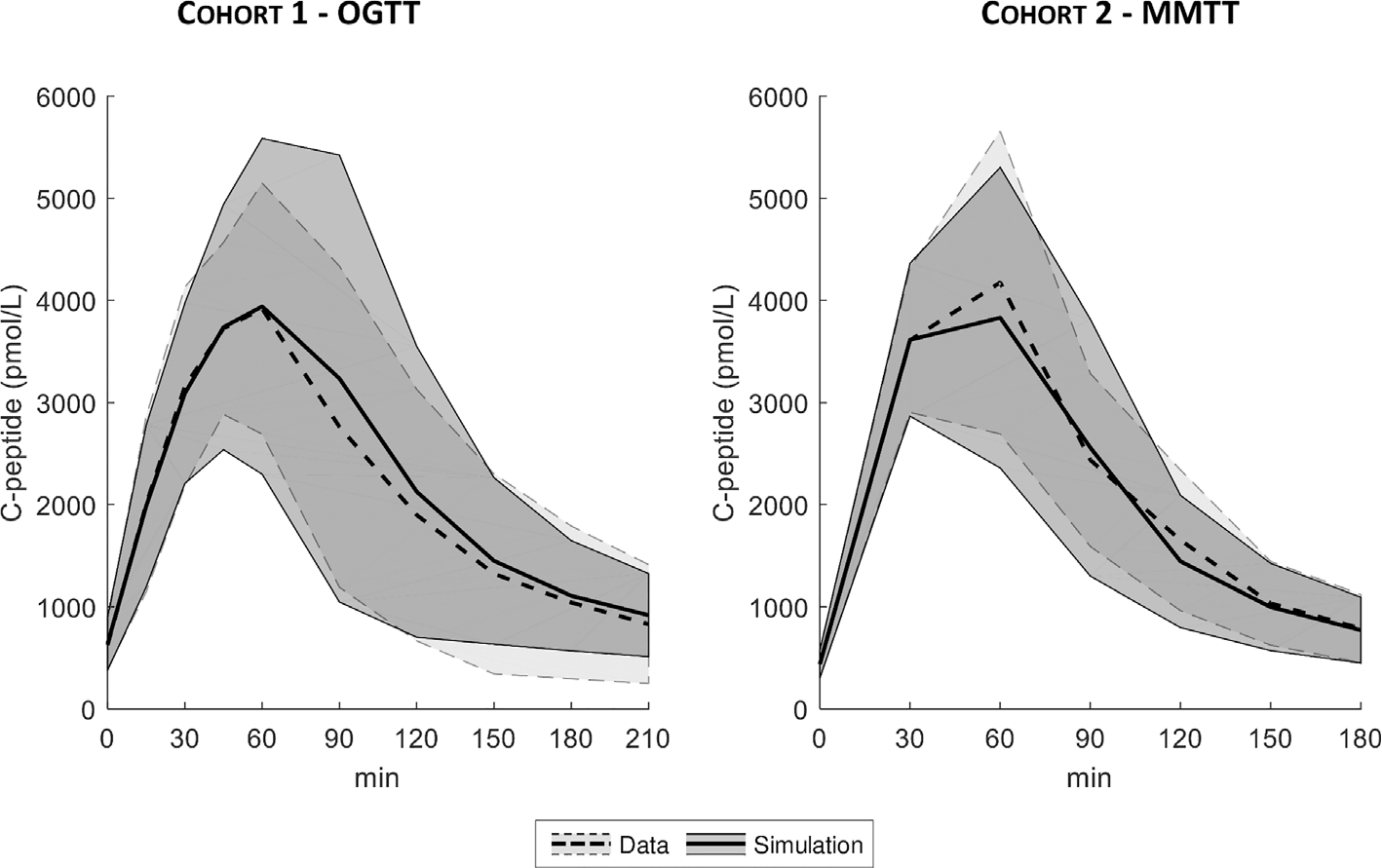
Mean ± SD of true (dashed line with light shaded area) vs. simulated data (continuous line with dark shaded area), for both Cohort 1- OGTT (left) and Cohort 2 - MMTT (right).

The distributions of the simulated vs. real C-peptide concentrations were very similar for both cohorts (**Figure 6**). Model parameters were estimated with precision in almost all the subjects with CV of 8 [5, 16] %, 19 [15, 24] %, and 17 [13, 25] % for the VC, DB, and PHH approach, respectively.

**Figure 7:**
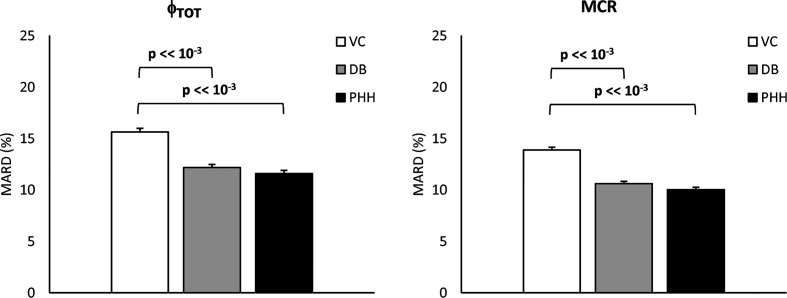
Mean Absolute Relative Difference (MARD) of estimated vs. true Φ_tot_ and MCR (left and right, respectively) for the VC, DB, and PHH approach calculated from the 1100 simulated C-peptide traces (mean ± SE).

The MARD of estimated vs. true Φ_tot_ and MCR are reported in **Figure 7**. MARD for Φ_tot_ was 14 [7, 22] % with the VC, 11 [5, 17] % with the DB and 9 [5, 16] % with the PHH approach. MARD for MCR was 13 [7, 20] % with the VC, 9 [5, 15] % with the DB and 8 [4, 14] % with the PHH approach. For both parameters, the Kruskal-Wallis test highlighted a significant difference in MARD among the identification approaches. The post-hoc analysis revealed a significant difference of VC vs. DB and PHH, but not between DB vs. PHH approaches. Moreover, the overall MARD, calculated by pooling all the estimated parameters, was 23 [11, 44] % vs. 14 [6, 24] % vs. 14 [6, 25] % with the VC vs. DB vs. PHH approach, respectively. Also in this case, Kruskal-Wallis test detected a significant difference between approaches which was confirmed by post-hoc analysis only when comparing VC vs. DB and PHH approach.

## Discussion

In this work, we tested the validity of the OCMM (11, 18) coupled with the Van Cauter population model for C-peptide kinetics in post-gastric bypass surgery individuals suffering from postprandial hyperinsulinemic hypoglycemia. We observed unsatisfactory results in terms of the model ability to predict the data (**Figure 4**). We hypothesized that this could be due to a mismatch between the actual C-peptide kinetics of the specific population under study and those predicted by the Van Cauter population model. To overcome this limitation, we used a Bayesian approach to estimate C-peptide kinetics from the data and tested the performance of the two different approaches using an *in silico* experiment.

While the OCMM prediction with the VC approach underestimated the C-peptide peak and overestimated the C-peptide tail, model fit of the data was satisfactory in all subjects with the DB approach (**Figure 4**). Our results, as illustrated by the higher C-peptide MCR with the DB vs. VC approach (**Figure 5** bottom), are suggestive of faster C-peptide kinetics in post-gastric bypass patients suffering from postprandial hyperinsulinemic hypoglycemia compared to values predicted by the Van Cauter population model (14).

The better model prediction achieved with the DB approach was expected since, unlike the VC approach, the C-peptide kinetic parameters are allowed to adapt to the specific data. However, this does not demonstrate that the estimated kinetic and secretion parameters are closer to the true ones. To assess the validity of the proposed approach, a simulation study was performed. Results showed that the MARD of estimated vs. true parameters was significantly lower with the DB vs. VC approach for both Φ_tot_ and MCR (**Figure 7**), suggesting that the DB approach, despite exploiting the *a priori* information derived from the VC model, allows to estimate model parameters closer to the true ones than the VC approach. Similar results were also obtained when using a *prior* better reflecting the characteristics of the population under study (PHH approach), indicating that final results are minimally affected by the specific choice of the *prior*.

A limitation of the present study is the use of only one of the possible C-peptide secretion models available in the literature (11) and we imputed the unsatisfactorily prediction of the data to a mismatch of C-peptide kinetics while this could also be due to inadequacy of the model for the specific population. Using other models, e.g., (8–10), could lead to different conclusions depending on model structure and *a priori/a posteriori* identifiability properties. However, the C-peptide secretion model adopted here (11) has been used in many studies either on different populations, e.g., healthy (28), prediabetes (29), and subjects with type 2 diabetes (30), and experimental conditions, e.g., testing the effects of pharmacological treatments (31, 32), while always showing its ability to describe the data with C-peptide kinetic parameters fixed to the ones predicted by the Van Cauter population model. Another limitation is that the proposed DB approach was validated in a simulation framework only, while a C-peptide bolus experiment in the same subjects or inclusion of a control group with previous assessment of C-peptide kinetics would be required. In other words, the higher C-peptide MCR suggested by our results need to be confirmed by the current gold-standard experiment using C-peptide bolus under somatostatin infusion. Nevertheless, we would like to point out that the methodology outlined in this work is applicable beyond the study of insulin secretion in this population. The described approach will allow to study insulin secretion also in other metabolic disorders possibly affecting C-peptide kinetics (e.g., renal diseases) or to study secretion of other hormones for which a population model of the kinetics is not yet available (e.g., glucagon). Noteworthy, as a first step to study this population, we focused on C-peptide secretion and kinetics only. However, assessing insulin kinetics and hepatic insulin extraction is also of great interest. To do so, we will apply this methodology to estimate insulin kinetics and hepatic insulin extraction in future works.

In conclusion, the results of the present study show the limitations of the OCMM coupled to the Van Cauter population model to accurately describe C-peptide data in post-gastric bypass individuals suffering from postprandial hyperinsulinemic hypoglycemia. Consequently, its validity to study insulin secretion and β-cell function in this population is limited. To overcome this limitation, we propose an alternative approach by estimating C-peptide kinetics from the data using a Bayesian approach. This opens new possibilities for the study of hormones for which population kinetic models are unavailable. Overall, our results suggest faster C-peptide metabolic clearance rate in post-gastric bypass individuals suffering from postprandial hyperinsulinemic hypoglycemia compared to previously studied populations. While the ability of the new approach to describe C-peptide data was tested *in silico*, further confirmation using *in vivo* experiments are warranted.

## Data Availability Statement

The original contributions presented in the study are included in the article. Further inquiries can be directed to the corresponding author.

## Ethics Statement

The studies involving human participants were reviewed and approved by Local ethics committee (Kantonale Ethikommission Bern, Bern, Switzerland for NCT03609632 and Ethikkommission Nordwest- und Zentralschweiz, Basel, Switzerland for NCT03200782). Study related procedures were performed in accordance with the local ethics standards and with the Declaration of Helsinki. The patients/participants provided their written informed consent to participate in this study.

## Author Contributions

All authors contributed to the article and approved the submitted version. MS performed the analysis, contributed to the discussion and wrote the manuscript. DH and LB provided the data, contributed to the discussion and writing the manuscript. MH and MD provided the data, contributed to the discussion, and edited the manuscript. CDM reviewed data analysis, contributed to results interpretation and writing the manuscript. CDM is the guarantor of this work and, as such, had full access to all the data in the study and takes responsibility for the integrity of the data and the accuracy of the data analysis.

## Funding

This work was supported by MIUR (Italian Minister for Education) under the initiative “Departments of Excellence” (Law 232/2016), University of Padova under the initiative “SID-Networking Project 2019”, the Swiss National Science Foundation (PCEGP3_186978) and the Scientific Fund of the Department of Diabetes, Endocrinology, Nutritional Medicine, Inselspital as well as the Diabetes Center Bern and the University Hospital in Basel.

## Conflict of Interest

The authors declare that the research was conducted in the absence of any commercial or financial relationships that could be construed as a potential conflict of interest.
